# Oxidative Stress and Neurodevelopmental Outcomes in Rat Offspring with Intrauterine Growth Restriction Induced by Reduced Uterine Perfusion

**DOI:** 10.3390/brainsci11010078

**Published:** 2021-01-08

**Authors:** Marcelo E. Rains, Colin B. Muncie, Yi Pang, Lir-Wan Fan, Lu-Tai Tien, Norma B. Ojeda

**Affiliations:** 1 Department of Pediatrics, University of Mississippi Medical Center, Jackson, MS 39216, USA; marcelorains@gmail.com (M.E.R.); ypang@umc.edu (Y.P.); lwfan@umc.edu (L.-W.F.); 2 Department of Surgery, University of Mississippi Medical Center, Jackson, MS 39216, USA; cmuncie@umc.edu; 3 School of Medicine, Fu Jen Catholic University, New Taipei City 24205, Taiwan

**Keywords:** intrauterine growth restriction, reduced uterine perfusion, motor and cognitive development, oxidative stress

## Abstract

Intrauterine growth restriction (IUGR) is a major cause of morbidity and mortality and is worldwide associated with delayed neurodevelopment. The exact mechanism involved in delayed neurodevelopment associated with IUGR is still unclear. Reduced uterine perfusion (RUP) is among the main causes of placental insufficiency leading to IUGR, which is associated with increases in oxidative stress. This study investigated whether oxidative stress is associated with delayed neurodevelopment in IUGR rat pups. Pregnant rats were exposed to RUP surgery on gestational day 14 to generate IUGR rat offspring. We evaluated offspring’s morphometric at birth, and neurodevelopment on postnatal day 21 (PD21) as well as markers of oxidative stress in plasma and brain. Offspring from dams exposed to RUP showed significant (*p* < 0.05) lower birth weight compared to controls, indicating IUGR. Motor and cognitive deficits, and levels of oxidative stress markers, were significantly (*p* < 0.05) elevated in IUGR offspring compared to controls. IUGR offspring showed significant (*p* < 0.05) negative correlations between brain lipid peroxidation and neurocognitive tests (open field and novel object recognition) in comparison with controls. Our findings suggest that neurodevelopmental delay observed in IUGR rat offspring is associated with increased levels of oxidative stress markers.

## 1. Introduction

Intrauterine growth restriction (IUGR) is the term attributed to a fetus that has not reached its full growth potential, as measured by weight, length, and head circumference [1]; it represents a condition now considered to have a major negative impact on global neonatal health and outcomes [2]. IUGR is estimated to occur in 5% to 7% of all pregnancies [3]. Factors leading to IUGR are associated with environmental, maternal, placental, and fetal disturbances [3]. However, the most common identifiable cause of IUGR is placental insufficiency [3]. Growth restricted babies have always existed. What has not always been available, however, is the technology to detect growth-restricted development in utero, and thence to help these neonates reach full growth potential. Such relatively recent technologies as ultrasound, which can measure biparietal diameter, head circumference, abdominal circumference and femur length, have become important tools in the effort to better characterize and address IUGR [4].

IUGR fetuses are more likely to have neurological impairments, which affect all domains of cognition and motor development [5,6]. In some cases, postnatal “catch-up” phenomenon is observed; however, it seems to be related to somatic growth, rather than including neurodevelopment improvement [7]. The effects on neurodevelopment are more permanent with impairments that profoundly affect the functional status of these infants across development. These impairments are associated with lower intelligence, poor overall academic performance, low social competence, and behavioral problems, including motor and psychological disorders, as well as long-term global cognitive deficiencies [2,8].

IUGR has been associated with a reduced uterine perfusion, which can occur, in pre-eclampsia [9]. Studies in the past decade have indicated that, in IUGR, a lessening of placental blood supply may result in placental ischemia, in turn triggering a cascade of inflammatory events [10]. Placental insufficiency as a cause of insufficient transformation of the spiral arteries into vessels of low resistance [11] is associated with uneven circulation, and inefficient oxygen and nutrient exchange which may increase degrees of hypoxemia and lactic acidemia leading to impairment of fetal blood flow and fetal growth restriction [12,13,14]. Placental insufficiency is associated with increased production of free radicals and vascular dysfunction, leading to alterations in oxidative metabolism and thereby increases fetal oxidative stress [15,16]. Moreover, several previous studies have shown brain susceptibility to oxidative stress and related brain disorders [15,17,18,19]. On a molecular level, oxidative stress causes brainstem mitochondrial dysfunction [18], as well as dysfunction in proteins, nucleic acids, and lipids [19]. Overall, disorders associated with increased oxidative stress in the brain include memory deficits [20,21], learning problems [22], cognitive behavior disorders [23], migraine headaches [24], motor deficits (cerebral palsy) [25], nocifensive behavior [26], schizophrenia [27] and Parkinson’s disease [28]. These findings suggest a correlation between oxidative stress and neurodevelopmental outcomes.

Investigating these mechanisms in an experimental model is important to help clarify the post-natal timeline and progression of motor function and cognitive development in offspring exposed to IUGR and increased oxidative stress in-utero. The present study extended previous experimental work by our lab that suggested associations between IUGR and oxidative stress and its effect on offspring health outcomes [29,30,31]. In this study, we investigated whether oxidative stress was associated with the neurodevelopmental delay observed in IUGR rat offspring.

## 2. Materials and Methods

### 2.1. Animals and Experimental Design

All experimental procedures were conducted in accordance with the National Institutes of Health guidelines. All experiments were approved by the Institutional Animal Care and Use Committee at the University of Mississippi Medical Center. Every effort was made to minimize the number of animals used and their suffering. Timed pregnant CD^R^ (Sprague Dawley) IGS (International Genetic Standardization Program) rats were purchased from Charles Rivers Laboratories International, Inc. (Raleigh, NC, USA). Animals were housed in a temperature-controlled room (22 ± 2 °C) with a cycle of 12 h of dark and 12 h of light with free access to food and water. Pregnant rats underwent either a reduced uterine perfusion (RUP) procedure or a sham procedure at gestational day 14 (E14). All pregnant rats delivered offspring at term. The gestation length of a Sprague-Dawley rat is between 21 and 23 days. The day of birth was defined as postnatal day 0 (PD0). The offspring from dams exposed to sham procedure were designated as the control group, and offspring from dams exposed to RUP surgery were designated as the IUGR group. Male and female offspring from control pregnant dams and reduced uterine perfusion pregnant dams were included in the study. To reduce the litter-related bias, we utilized 1 male and 1 female from each litter. We utilized 48 animals for this study, with 32 offspring distributed as Control and IUGR, male and female *n* = 8 per group, and 16 dams distributed equally for RUP and Sham, *n* = 8 per group. Animals were evaluated at birth (PD0) and at PD7, PD14 and PD21.

### 2.2. Reduced Uterine Perfusion (RUP) Procedure in the Pregnant Rat

Pregnant rats underwent either a reduced uterine perfusion (RUP) procedure or a sham procedure at gestational day 14 (E14) as described previously with modifications [29,30,31]. Briefly, animals underwent anesthesia with isoflurane gas, followed by opening of the abdominal cavity via a ventral midline incision. A silver vascular clip (0.203 mm ID) was slipped around the lower abdominal aorta above the iliac bifurcation and below the renal arteries. Additionally, two silver clips (0.100 mm ID) were placed on both uterine tributary branches of ovarian arteries. Control pregnant rats underwent a sham RUP procedure that included opening the abdominal cavity, identification and counting of offspring inside uterine horns, without placing silver vascular clips. The two muscular layers and the skin incision were closed using a 3.0 silk suture. Other investigators reported a reduction of approximately 40% in blood flow to the placentae with this method [10,32].

### 2.3. Behavioral Testing

#### 2.3.1. Grip Strength Assessment

Behavioral tests were performed as described previously with modifications [33]. To test motor coordination, grip strength assessment (GSA) was performed at PD7, PD14 and PD21. The equipment consisted of a metal string of 50 cm length pulled taut across two supports and elevated 40 cm above a flat surface. Each offspring’s front paws were placed on the metal string and evaluated according to a standardized scale. The scale scores were 0: fall off; 1: hangs onto string by two forepaws; 2: while hanging with both forepaws, the offspring tries to climb on the string; 3: hangs onto the string by two forepaws plus one or both hind paws; 4: hangs onto string by all four paws plus tail wrapped around string, 5: escapes by walking on the string. They were given 3 tries each, and the attempts for each pup were averaged and analyzed.

#### 2.3.2. Open Field Test

An open field test (OFT) was performed at PD21 to assess exploratory behavior [33] using locomotor activity monitors (Columbus Automex, Columbus, OH, USA). The offspring were acclimated to the room before testing. Animals were placed individually in the center (20 × 20 cm) of a chamber (40 × 40 cm) with clear Plexiglas walls and floors located within the Columbus Automex units. The observation method was automated using infrared beam grid detection. Time spent in the center and periphery of the apparatus was recorded for 30 min. The duration of the time spent in the middle quadrants was compared to the time spent in the periphery. Results are reported as time spent in the central area of the box.

#### 2.3.3. Novel Object Recognition

A novel object recognition (NOR) test was performed at PD21 using the Columbus Automex Activity monitors and following a modified method previously reported [34]. The software recorded frequency of entries and the duration of time spent in each of the monitor’s 16 squares defined by infrared beams. The software counted the beam breaks and duration time spent in each square. The time spent around the novel object was compared between the IUGR and control groups. Animals were allowed to acclimate for 60 min in the room where the NOR chamber was located. The test included the following phases: (1) Exploration Trial, a 20–30 min acclimation phase in the Automex Activity chamber (40 × 40 cm) during which locomotor activity and location were monitored. At the end of this phase, animals were retired from the chamber for 5 min before starting the next phase; (2) First Interaction Phase, two objects (Nalgene covered blocks of lead) were randomly placed on any two diagonal corners of the chamber. The animal was allowed to freely explore the area and the objects for 5 min; (3) Inter-Trial Interval (ITI), a five minutes break when animals were removed from the chamber after the first interaction phase. During this phase, animals were placed on their respective cages, and both objects were removed from the chamber, lightly cleaned, and dried. One of the objects from the first interaction was returned to the chamber to the same place, and a novel object was placed in the other corner; (4) Second Interaction Phase, a five minutes interaction phase in which the animal was returned to the familiar chamber and monitored as before. After the test was completed, the animal was returned to the home cage and the entire apparatus was thoroughly cleaned before beginning the next run. Once all trials finished, the animals were returned to the housing facility. Each animal participated one time in each phase and ITI.

### 2.4. Determination of Oxidative Stress

#### 2.4.1. Determination of Plasma Oxidative Markers

The levels of plasma superoxide anion, NADPH oxidase dependent superoxide anion, extracellular superoxide dismutase (E-SOD) activity and the total antioxidant capacity (TAC) were performed as described previously with modifications [29] and measured with microplate assay kits (Cayman Chemical, Ann Arbor, MI, USA). A blood sample from each offspring was collected immediately after decapitation with a sharp guillotine on PD21. Blood was then centrifuged at 1000× *g* for 10 min at 4 °C. The supernatant was decanted, labeled, and stored at −80 °C until sample analysis. The assay was performed according to the manufacturer’s instructions, and data were acquired using a 96-well plate reader (Bio-Tek Instruments, Inc., Winooski, VT, USA).

#### 2.4.2. Measurement of Brain Lipid Peroxidation

Lipid peroxidation was determined in PD21 brain and plasma samples by measuring malondialdehyde (MDA) levels as thiobarbituric acid-reactive substances (TBARS) [35]. Whole brain tissue from each offspring was harvested, placed in aluminum foil, snapped frozen in liquid nitrogen and then placed in −80 °C for storage. The whole brain was homogenized in RIPA buffer containing a protease inhibitor cocktail (Roche Pharmaceuticals) for TBARS assays. The assay was performed according to manufacturer’s instructions (Cayman Chemical, Ann Arbor, MI, USA), and data were acquired using a 96-well plate reader (Bio-Tek Instruments, Inc., Winooski, VT, USA).

### 2.5. Statistics

GraphPad 6 and SPSS 22 statistical software were utilized to perform data analysis of the results. Student’s *t*-test was used to find statistical significance between the two groups for the behavioral tests. The two-way ANOVA with multivariate analysis was used to calculate differences between groups. A Pearson Correlation Coefficient was used to measure the association between brain lipid peroxidation and open field test, and between brain lipid peroxidation and novel object recognition. Results are reported as mean ± SEM, statistical significance was set at *p* < 0.05, with a statistical power of at least 0.85.

## 3. Results

### 3.1. Effects of Reduced Uterine Perfusion on Offspring’s Body Weight

**Figure 1:**
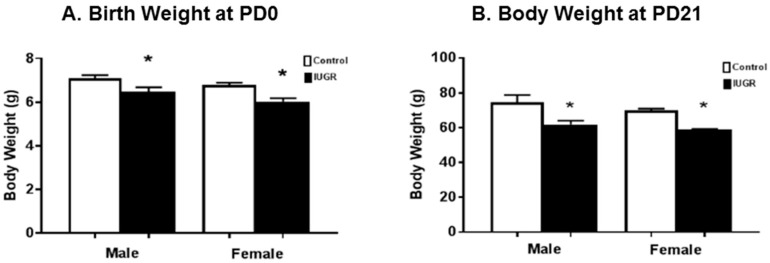
Offspring’s body weights at birth (postnatal day 0 (PD0)) and at postnatal day 21 (PD21). Male and female offspring exposed to reduced uterine perfusion (RUP), presented in the black columns, show significant reductions in (**A**) birth weight (PD0), and (**B**) body weight at PD21 compared to offspring exposed to sham surgery presented in the white columns. All groups: *n* = 8/sex. * *p* < 0.0001 vs. control group. Graphs represented as mean ± SEM.

Both male and female rat offspring exposed to RUP showed significantly lower weight at birth (PD0) (*p* < 0.0001) and at PD21 (*p* < 0.0001) compared to offspring exposed to sham surgery (Controls) (Figure 1A,B). These RUP-exposed offspring are considered intrauterine growth restricted (IUGR) based on their birth weights, which are at or below the 10th percentile of the birth weights in the control group [4]. There were no significant differences between sexes in the same group.

### 3.2. Behavioral Test in Offspring from Dams Exposed to RUP

#### 3.2.1. Grip Strength Assessment

**Figure 2:**
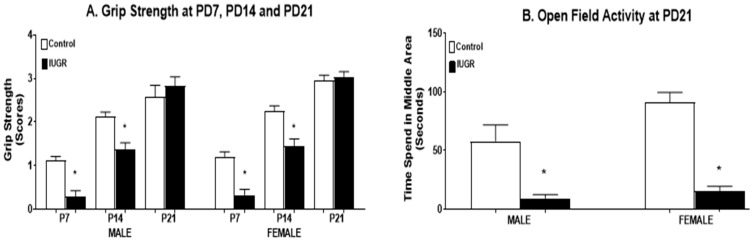
Motor behavioral assessment at PD7, PD14 and PD21. Male and female offspring exposed to RUP presented in the black columns as intrauterine growth restriction (IUGR), show significant reduction in (**A**) grip strength at PD7 and PD14, and (**B**) open field activity at PD21 compared to their counterparts exposed to sham surgery presented in the white columns as controls. All groups: *n* = 8/sex. * *p* < 0.0001 vs. control group. Graphs represented as mean ± SEM.

Motor coordination was determined by grip strength assessment [33] in IUGR and control offspring. There was a statistically significant (*p* < 0.0001) decrease in grip strength at PD7 and PD14 in the IUGR group versus the control group. However, by PD21, the IUGR offspring caught up to their control counterparts and had equal grip strength (Figure 2A). There were no sex differences seen in either IUGR or control offspring.

#### 3.2.2. Locomotor Activity

The open field test was used to determine locomotor behavior [33]. IUGR offspring, both male and female, showed a statistically significant (*p* < 0.0001) decrease in locomotor activity in the open field test at PD21 when compared to control groups (Figure 2B). No sex differences were seen in either IUGR or control offspring.

#### 3.2.3. Novel Object Recognition

**Figure 3:**
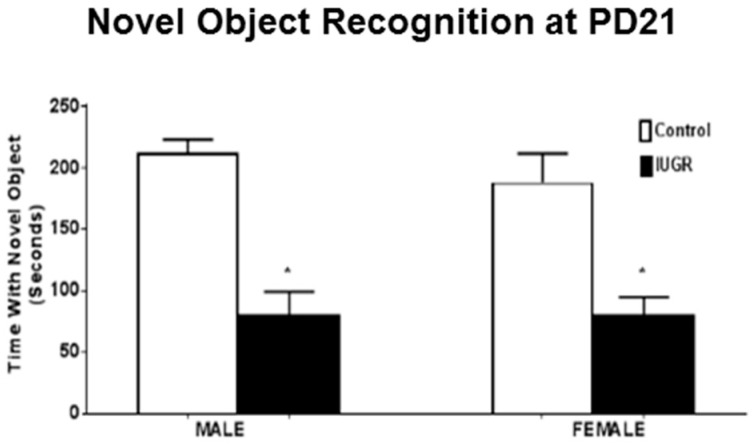
Cognitive behavior assessment at PD21. Male and female offspring exposed to RUP, presented in the black columns as IUGR, show significant reduction in novel object recognition compared to their counterparts exposed to sham surgery presented in the white columns as controls. All groups: *n* = 8/sex. * *p* < 0.0001 vs. control group. Graphs represented as mean ± SEM.

Cognitive behavior was determined by novel object recognition test [34]. Both male and female IUGR offspring showed a statistically significant (*p* < 0.0001) decrease in the time spent with novel object when compared to their control counterparts (Figure 3). No sex differences were seen in either IUGR or control offspring.

### 3.3. Molecular Measurements of Oxidative Stress

#### 3.3.1. Pro-Oxidation Markers

**Figure 4:**
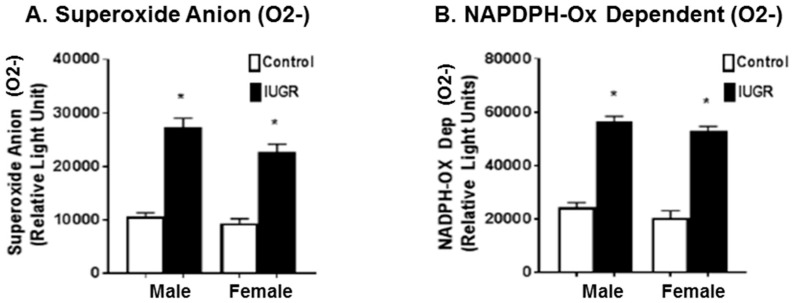
Pro-oxidation markers in plasma samples. Male and female offspring exposed to RUP, presented in the black columns as IUGR, show significant increase in pro-oxidation markers, (**A**) Superoxide Anion (O2^−^), and (**B**) Nicotinamide adenine dinucleotide phosphate-oxidase (NADPH-Ox) dependent O2^−^ when compared to their counterparts exposed to sham surgery presented in the white columns as controls. All groups: *n* = 8/sex. * *p* < 0.0001 vs. control group. Graphs represented as mean ± SEM.

Superoxide anion (O2^−^) and NADPH oxidase dependent superoxide anion (O2^−^) are short-lived radicals of molecular oxygens that are implicated in oxidative stress damage. Once formed, superoxide anions attack cellular components causing damage to lipids, proteins, and DNA [36,37]. At PD21, both male and female IUGR offspring showed a statistically significant (*p* < 0.0001) increase in plasma levels of superoxide anion (Figure 4A), and NADPH oxidase dependent superoxide anion (Figure 4B) as compared with the control group. No sex differences in plasma levels of superoxide anion and NADPH oxidase-dependent superoxide anion were observed in IUGR or control offspring.

#### 3.3.2. Anti-Oxidative Stress Markers

**Figure 5:**
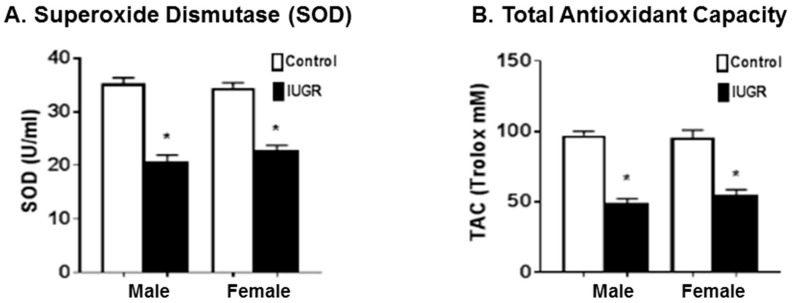
Markers of anti-oxidative stress in plasma samples. Male and female offspring exposed to RUP, presented in the black columns as IUGR, show significant decrease in antioxidant markers, (**A**) superoxide dismutase (SOD) activity, and (**B**) total antioxidant capacity when compared to their counterparts exposed to sham surgery presented in the white columns as controls. All groups: *n* = 8/sex. * *p* < 0.0001 vs. control group. Graphs represented as mean ± SEM.

Antioxidants play an important role in preventing the formation and scavenging of free radicals and other potentially toxic oxidizing species. The enzyme superoxide dismutase (SOD) suppresses the activity of the superoxide anion by converting O2^−^ to hydrogen peroxide. Total antioxidant capacity assay was used to measure the non-enzymatic antioxidant capacity of small molecule antioxidants, which provide an indication of the overall capability to counteract reactive oxygen species (ROS) [36,37]. At PD21, IUGR offspring, both male and female showed a statistically significant (*p* < 0.0001) decrease in plasma levels of SOD activity (Figure 5A) and total antioxidant capacity (Figure 5B) as compared to their control counterparts. No sex differences in plasma levels of SOD and total antioxidant capacity were observed in IUGR or control pups.

#### 3.3.3. Lipid Peroxidation

**Figure 6:**
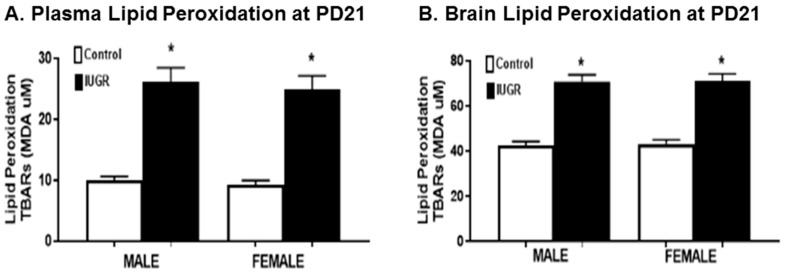
Lipid peroxidation in plasma and brain samples. Male and female offspring exposed to RUP, presented in the black columns as IUGR, show significant increase in levels of lipid peroxidation in (**A**) plasma samples, and (**B**) brain tissues when compared to their counterparts exposed to sham surgery presented in the white columns as controls. All groups: *n* = 8/sex. * *p* < 0.0001 vs. control group. Graphs represented as mean ± SEM.

To investigate the effects of RUP exposure on oxidative stress, lipid peroxidation was assessed in plasma and brain tissues by measuring thiobarbituric acid-reactive substances (TBARS) [35]. At PD21, IUGR offspring, both male and female, showed statistically significant (*p* < 0.0001) increased levels of plasma lipid peroxidation (Figure 6A) and brain lipid peroxidation (Figure 6B) when compared to control counterparts. There were no sex differences seen in either IUGR or control offspring.

### 3.4. Correlation between Molecular Markers and Behavioral Tests

**Figure 7:**
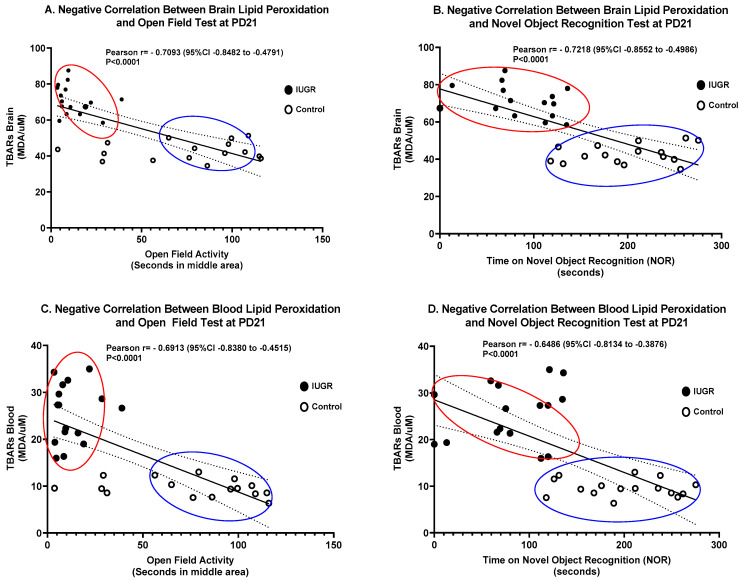
Correlations between levels of lipid peroxidation in brain and blood and neurodevelopment tests. There were negative correlations between brain lipid peroxidation and (**A**) open field activity (Pearson coefficient r = −0.7093; 95% CI = −0.8482 to −0.4791; *p* < 0.0001), and (**B**) novel object recognition (Pearson coefficient r = −0.7218; 95% CI = −0.8552 to −0.4986; *p* < 0.0001), and between blood lipid peroxidation and (**C**) open field activity (Pearson coefficient r = −0.6913; 95% CI = −0.8380 to −0.4515; *p* < 0.0001) and (**D**) novel object recognition (Pearson coefficient r = −0.6466; 95% CI = −0.8134 to −0.3876; *p* < 0.0001). Controls are presented as white dots and IUGR as black dots. All groups: *n* = 8/sex.

Correlations between levels of lipid peroxidation and neurobehavioral development tests were evaluated in the current study. Interestingly, we found a statistically significant negative correlation between levels of lipid peroxidation in the brain and locomotor activity performance in the open field test (*p* < 0.0001) (Figure 7A), and cognition in the novel object recognition test (*p* = 0.0001). Similarly, we found a statistically significant negative correlation between levels of lipid peroxidation in blood and locomotor activity performance in the open field test (*p* < 0.0001) (Figure 7C), and cognition in the novel object recognition test (*p* = 0.0001) (Figure 7D). IUGR offspring with the lowest performance rates showed the highest levels of lipid peroxidation in the brain, and in blood (Figure 7A–D). There were no sex differences seen in either IUGR or control pups when comparing male to female rat pups.

## 4. Discussion

Consistent with our previous study [29,38], both male and female rat offspring exposed to RUP showed significantly lower weight at birth (PD0) (Figure 1A). The birth weights of RUP-exposed offspring were below the 10th percentile for birth weights in the control group; therefore, these RUP-exposed offspring are considered IUGR, similar to the most widely used definition of this condition in humans [39]. Placental insufficiency (RUP) also affected neurodevelopmental outcomes including sensorimotor behavior in IUGR offspring. The grip strength assessment showed a decrease in grip strength in IUGR offspring at PD7, PD14, but not at PD21 in comparison with control offspring (Figure 2A). This time-related discrepancy could be explained by somatic “catch up” phenomenon in IUGR rat pups reported by others [7], or due to differences in body weight between IUGR and control offspring at PD21. IUGR offspring were lighter and could be able to pull their weight up more easily compared to their control counterparts.

The RUP exposure also caused long-term developmental (Figure 1B), locomotor (Figure 2B) and cognitive dysfunction in juvenile rats at PD21 (Figure 3). The open field test showed a significant difference between control and IUGR offspring. IUGR offspring tended to prefer the periphery and did not show the same level of activity observed in the control group. The open field test often is used to measure the level of anxiety and motor activity in the rats [40]. The changes shown by IUGR offspring in this test could represent signs of delayed neurodevelopment as previously reported in a mouse model of motor abnormalities and cognitive deficits [41]. Alterations in the behavioral elements of the open field test, such as shorter distance travelled in the central zone, less time spent in the central zone, and more time spent in the corner (periphery zone), are associated with anxiety [42,43]. The novel object recognition showed that IUGR offspring spent less time exploring the novel object compared to their control counterparts. This could mean memory or spatial recognition deficits in the IUGR offspring when compared to the control group [44].

It has been reported that preeclampsia-related IUGR, which is associated with more severe placental oxidative stress, results in a higher impact on developmental outcomes and the additional release of pro-inflammatory factors when compared with unexplained IUGR [4,16]. Cells are normally able to balance the production of oxidants and anti-oxidants to maintain redox equilibrium; however, oxidative stress occurs when this equilibrium is lost by excess levels of oxidants, or depletion of anti-oxidants [36,37]. Our previous studies indicate that rat offspring exposed to decreased uterine perfusion showed increased renal oxidative stress and ischemic renal injury susceptibility [29,30,38]. In the current study, our results show that the levels of pro-oxidative stress markers, including superoxide anion and NADPH oxidase-dependent superoxide anion, were higher in the plasma samples of IUGR offspring compared to controls (Figure 4), while the levels of anti-oxidative markers, including SOD and total antioxidant capacity, were lower in IUGR rats as compared to their control counterparts (Figure 5). Thus, our findings indicate an overall trend of increased oxidative stress in rat offspring exposed to decreased uterine perfusion and suggest that the antioxidant system could be depleted due to increased levels of free radicals in IUGR offspring. The exposure to RUP could lead to reductions in the availability of oxygen and nutrients for the fetuses resulting in IUGR offspring with increased oxidative stress levels [16]. As a result of increased oxidative stress, the levels of plasma and brain lipid peroxidation were increased in IUGR offspring at PD21 (Figure 6). The phospholipids in the mammalian brain membranes are extremely sensitive to free radicals, which help to maintain a fine metabolic equilibrium [45]. However, the excessive generation of free radicals can result in brain lipid peroxidation with detrimental effects on cells function [45]. Brain lipid peroxidation is associated with conditions such as brain injury, cerebral ischemia, Alzheimer’s Disease, and Autism Spectrum Disorders [46]. Consequently, the functional involvement of lipid peroxidation in IUGR related neurodevelopmental delay is strongly suggested based on previous reports [45,46], and the findings in this study.

Previous studies reported that elevated levels of oxidative stress result in detrimental effects on neurodevelopment in premature and IUGR newborns [47,48]. The brain is susceptible to oxidative stress and the selective vulnerability of neurons to free radicals is also found in the brain [37,49]. The CA1 regions of the hippocampus, cerebellar granule neurons, and substantia nigra pars compacta (A9) are very sensitive to oxidative stress [37,49]. The gray matter also shows vulnerability to free radicals in IUGR in preterm infants [49]. Therefore, motor and cognitive neurodevelopmental outcomes may be impacted by elevated levels of oxidative stress. Other investigators utilizing similar rat models of IUGR or placental ischemia reported many structural changes in the hippocampus along with deficits on learning, memory, social interactions and sensorimotor abilities [50,51]. Moreover, the effects of intrauterine hypo perfusion or prenatal ischemia on neuroanatomical and functional organization of sensorimotor systems and behavioral and cognition were reported in several publications by others [52,53].

Our results show that RUP exposure is associated with IUGR, increased oxidative stress, and long-term locomotor and cognitive dysfunction in juvenile rats at PD21. Maternal molecular hydrogen treatment, which scavenges hydroxyl radicals, has been reported to decrease RUP-induced fetal oxidative stress and hippocampal damage in PD7 rats [54,55]. Our previous studies also have shown that chronic treatment with an antioxidant tempol normalized renal markers of oxidative stress in IUGR offspring [29,38]. Future studies are needed to investigate whether reduction in oxidative stress markers by an antioxidant treatment is correlated with improvements on neurodevelopment outcomes in our model of IUGR rat offspring.

IUGR fetuses may have short-term and long-term neurodevelopmental impairments [5,6], which typically lead to lower intelligence, overall poor academic performance, low social competence, and behavioral problems including motor and psychological disorders, as well as long-term global cognitive impairments [2,8]. The experimental model utilized in this study mimicked the human condition of exposure to reduced uterine perfusion resulting in IUGR babies with neurodevelopmental dysfunction. Moreover, the inverse correlation between levels of brain lipid peroxidation (TBARS) and scores in the open field activity and the test of novel object recognition strongly suggests that oxidative stress may be involved in the neurodevelopmental impairment observed in IUGR offspring in this study (Figure 7A,B). Shoji et al. have reported that in very low birth weight infants, 8-hydroxy-2″-deoxyguanosine (8-OHdG), a urinary oxidative DAN damage marker, correlated with mental development in 18-month-old neonates [47]. In addition, preeclampsia-induced low maternal total antioxidant status levels are associated with poor neuro-motor outcomes in 1-year-old neonates [48].

## 5. Conclusions

In summary, our findings suggest that early exposure to RUP is associated with development of IUGR, neurodevelopmental impairments, increased brain lipid peroxidation, and plasma oxidative stress in juvenile rat offspring. Further studies are warranted to test causality effects of oxidative stress on neurodevelopmental outcomes. Moreover, future studies can explore antioxidant treatments to investigate whether the reduction in oxidative stress markers is correlated with improvements on neurodevelopment outcomes in this rat model of IUGR offspring. In addition, our results contribute to the advance in our knowledge of the correlation of RUP exposure-induced IUGR, oxidative stress and neurodevelopment outcomes.

## Data Availability

The data presented in this study are available on request from the corresponding author. The data are available following Institutional and Federal guidelines requesting the use of a data-sharing agreement to impose appropriate limitations on users, to ensure data security at the recipient site and to prevent data manipulation.
